# Differential MRI atrophy profiles and incident dementia: A cross-national comparison

**DOI:** 10.1177/13872877251371734

**Published:** 2025-09-03

**Authors:** Monica E Walters, Brent J Small, Ross Andel, Ondrej Lerch, Hana Horakova, Victor A Molinari, Jason L Salemi, Martin Vyhnalek, Zuzana Nedelska, Jakub Hort, for the Alzheimer’s Disease Neuroimaging Initiative

**Affiliations:** 1 Department of Psychology, University of Michigan, Ann Arbor, MI, USA; 2 School of Nursing, The University of North Carolina at Chapel Hill, Chapel Hill, NC, USA; 3 Edson College of Nursing and Health Innovation, Arizona State University, Phoenix, AZ, USA; 4 Department of Neurology, Second Faculty of Medicine, Charles University and Motol University Hospital, Praha, Czech Republic; 5 School of Aging Studies, University of South Florida, Tampa, FL, USA; 6 College of Public Health, University of South Florida, Tampa, FL, USA; 7 International Clinical Research Center, St. Anne's University Hospital and Faculty of Medicine, Masaryk University, Brno, Czech Republic

**Keywords:** Alzheimer's disease, cognitive dysfunction, dementia, latent class analysis, magnetic resonance imaging, prospective studies, survival analysis

## Abstract

**Background:**

Alzheimer's disease (AD) is a progressive neurodegenerative disorder with extensive neuropathological and clinical heterogeneity.

**Objective:**

We assessed empirically derived brain atrophy profiles in relation to incident AD dementia.

**Methods:**

A secondary data analysis of two prospective cohort studies was conducted, including participants without dementia from the Alzheimer's Disease Neuroimaging Initiative (ADNI; *n* = 1703) and the Czech Brain Aging Study (CBAS; *n* = 385). Latent profile analysis identified profiles across 10 pre-selected, AD-related brain regions derived from structural magnetic resonance imaging (hippocampus, middle temporal, superior temporal, precuneus, anterior cingulate, medial orbitofrontal, pericalcarine, precentral, lingual, caudate regions). Cox proportional hazards regression assessed how profiles related to incident AD dementia.

**Results:**

Four profiles emerged in ADNI: Minimal (*n* = 192), Mild (*n* = 691), Moderate (*n* = 567), and Severe (*n* = 253) Atrophy. Two profiles emerged in CBAS: Mild (*n* = 208) and Severe (*n* = 177) Atrophy. In ADNI, participants with Mild (HR = 3.11, 95% CI [1.43, 6.78]), Moderate (HR = 7.58, 95% CI [3.45, 16.68]), and Severe (HR = 16.95, 95% CI [7.39, 39.86]) Atrophy (versus Minimal) had increased incident AD dementia risk. In CBAS, participants with Severe Atrophy (versus Mild) had increased incident AD dementia risk (HR = 3.51, 95% CI [2.14, 5.77]). Controlling for baseline cognition attenuated effects for Mild (ADNI) and Severe (CBAS) Atrophy to non-significance.

**Conclusions:**

In two geographically and culturally distinct samples, magnitude of atrophy, not pattern across regions, determined classification into profiles, which predicted incident AD dementia. Findings highlight generalized, rather than region-specific, atrophy patterns associated with AD, and underscore the clinical utility of brain volumetry in identifying those with elevated incident AD dementia risk.

## Introduction

Over 57 million people worldwide had dementia in 2019, with numbers expected to nearly triple by 2050.^1^ Alzheimer's disease (AD) contributes to approximately 60–80% of dementia cases.^2^ With the advent of new treatment options addressing early symptomatic AD,^3,4^ better understanding of the underlying disease process and of factors associated with AD progression continues to be a prime research target.

One issue that prevents swifter progress in this area is the considerable heterogeneity in AD clinical phenotype across patients. Therefore, accurate and reliable characterization of AD remains a crucial aspect of AD research. To characterize variability in AD neuropathology, including atrophy patterns, some researchers have used data-driven approaches, including latent class analysis or other clustering techniques^5–11^. Data-driven methodologies look to identify quantitatively distinguishable subgroups to explain variability between people,^12^ thus presumably providing a more nuanced picture of variable atrophy profiles.

Much research on AD subtype classification includes participants with prevalent dementia (see for review^7,13^). Fewer studies include participants without dementia, whereby distinct subgroups based on atrophy patterns can be indicative of underlying future dementia risk.^6,10^ Among the few such studies, 3–4 neuropathology or atrophy subtypes emerged among older adults with amnestic mild cognitive impairment (MCI)^6,10^ and normal cognition,^9^ with results suggesting that individuals classified into profiles with more extensive pathology/atrophy had faster cognitive decline^9^ or higher dementia risk.^6,10^ These studies^6,9,10^ represent independent assessments of a single dataset (the Alzheimer's Disease Neuroimaging Initiative [ADNI]) which limits the generalizability of findings.

We built on previous research by characterizing brain atrophy subtypes in individuals initially without dementia using two datasets from geographically, culturally, and sociodemographically distinct regions (US: ADNI and Central Europe: Czech Brain Aging Study [CBAS^14^]), a rich set of presumed AD risk factors, and a comprehensive assessment of brain volumetry within this prospective study, allowing us to attempt replication of ADNI results with CBAS within one study. We aimed to characterize brain atrophy profiles derived from regional volumetric analyses and assess their predictive value for the risk of incident Alzheimer's dementia. Based on previous research,^6^ we hypothesized that 3–4 profiles would emerge in each dataset from normal volume to significant atrophy, and we expected that profiles with more extensive atrophy would exhibit higher risk for incident AD dementia compared to profiles with less extensive atrophy. AD is known to exist along a continuum^15^ with variability in atrophy levels across seemingly homogenous diagnostic groups.^16–18^ Specifically, some participants with normal cognition (or MCI) were found to have magnitude of atrophy equivalent to the average of participants with AD dementia, or magnitude of atrophy less than what would be expected given their clinical diagnosis.^6,9,10^ In addition, individuals with MCI convert to having normal cognition about as often as they convert to dementia.^19–21^ Therefore, we included participants across the AD continuum (i.e., normal cognition, subjective cognitive decline [SCD], MCI) in the analyses to determine how the latent subgroups of brain atrophy may characterize risk for incident AD dementia separate from clinical stage.

## Methods

We conducted a secondary data analysis of two prospective cohort studies, quantifying profiles in one and attempting to validate the profiles in the other. The study was approved by the University of South Florida's Institutional Review Board (IRB) and respective IRBs for ADNI and CBAS. Participants in both studies provided informed consent. All variable creation and analyses were conducted separately in the two datasets, thus representing a coordinated data analytic technique and a prime opportunity to assess validation of patterns across two geographically, culturally, and sociodemographically distinct cohorts.

### Participants

#### ADNI

ADNI is a longitudinal cohort study of individuals aged 55–90 years at baseline with normal cognition, subjective memory concern, MCI, or AD dementia from 63 sites in the United States and Canada.^22^ ADNI was started in 2003 as a public-private partnership under the direction of Dr Michael W. Weiner.^22^ Initial goals of ADNI were to standardize methodology used to assess the progression of AD dementia by including various types of data such as neuroimaging, clinical, neuropsychological, genetics, and biomarkers.^23^ Inclusion criteria into ADNI have been previously described.^24^ Participants are assessed every six months.^24^ For up-to-date information, see https://adni.loni.usc.edu/about/.

Data from ADNI were downloaded on November 30, 2022. There were 2406 participants from ADNI that had baseline data. Participants were excluded if: they had dementia at baseline (*n* = 414); they were missing data on sociodemographic characteristics (*n* = 5); they were missing all magnetic resonance imaging (MRI) data (*n* = 89), had <8 of the 10 volumetric regions of interest (ROIs) available (*n* = 5), failed MRI quality control or had hippocampus data only (*n* = 28); or had <2 study visits (*n* = 162), leaving an analytic sample of 1703 participants.

#### CBAS

CBAS is a two-site, clinic-based longitudinal cohort study started in 2005 recruiting nondemented older adults referred for cognitive difficulties perceived by the patients or their informants to assess patterns of cognitive decline and risk factors for dementia. We included all available data from the Prague CBAS site. Participants are included if they are 55 + years old and dementia-free at initial assessment.^14^ Additional inclusion criteria for CBAS have been previously described.^14^ Participants complete cognitive, biological, clinical, and neuroimaging assessments about annually.

Data from CBAS were received on October 28, 2022. The original CBAS sample included 1530 participants with SCD or MCI. Participants were excluded if they had more than six months between their MRI and cognitive assessments (*n* = 50), were missing MRI data (*n* = 510), or dates for MRI and/or cognitive assessments (*n* = 10), or had <8 of the 10 volumetric ROIs available (*n* = 139); were missing sociodemographic characteristics (*n* = 28); had <2 study visits (*n* = 379); progressed to a non-AD dementia (*n* = 24); or were missing date of dementia diagnosis (*n* = 5). The final analytic sample included 385 participants.

### Measures

#### AD dementia incidence

To diagnose dementia, ADNI uses the National Institute of Neurological and Communicable Disorders and Stroke and the Alzheimer's Disease and Related Disorders Association (NINCDS/ADRA) criteria^25^ and CBAS uses the Diagnostic and Statistical Manual of Mental Disorders (DSM)-IV-TR.^26^ AD dementia etiology is determined through the same clinical criteria in both ADNI and CBAS.^25^ Time to dementia was calculated in years as the difference between the baseline assessment and the first date of a dementia diagnosis.

#### Structural MRI

MRI scans for ADNI were conducted on both 1.5 T and 3 T scanners. Scan acquisition information for ADNI is available through their website (https://adni.loni.usc.edu/), and we used their provided, pre-computed FreeSurfer csv files. MRI images in CBAS were acquired on a Siemens Avanto 1.5 T scanner (Siemens AG, Erlangen, Germany) with a 12-channel phased-array head coil using high-resolution three-dimensional T1-weighted (3D T1w) Magnetization-Prepared Rapid Gradient Echo (MPRAGE) sequence with the following parameters: repetition time (TR)/echo time (TE)/inversion time (TI) = 2000/3.08/1100 ms, flip angle = 15°, 192 continuous partitions, slice thickness = 1.0 mm, and in-plane resolution = 1 mm. All acquisitions were visually inspected by a neuroradiologist to exclude participants with tumors, cortical infarcts, hydrocephalus, or other major brain pathology, and by a single CBAS researcher for quality control (e.g., excessive motion, sufficient coverage, etc.). Solely acquisitions deemed as having a sufficient technical quality were included.

Cortical reconstruction and volumetric segmentation of MRI images in both ADNI and CBAS was performed with the automated FreeSurfer image analysis suite (CBAS: version 5.3; ADNI: versions 4.3, 5.1, and 6.0; http://surfer.nmr.mgh.harvard.edu).^27^ MRI volumetry was measured in mm^3^ at baseline. Ten brain regions were used, based on past research^6^ that sought to reduce the number of regions in analyses by conducting a principal factor analysis: specifically, we include the hippocampus, middle temporal, superior temporal, precuneus, anterior cingulate, medial orbitofrontal, pericalcarine, precentral, lingual, and caudate regions. Although the frontal operculum was used as a ROI in prior work,^6^ we chose to include the pericalcarine region instead because (a) this region is segmented in CBAS (whereas the frontal operculum is not) and (b) it had the second highest factor loading (0.80) for the ROI aligning with the frontal operculum and should represent the region adequately. For the anterior cingulate cortex, both the rostral and caudal regions were included by adding these volumes together.

To calculate the ROIs, first, bilateral brain regions were calculated by adding the right and left volumes together. Then, these ROIs were adjusted for estimated intracranial volume (eTIV) by a residual methodology^28,29^ with the following formula: volume adjusted_i_ = volume raw_i_ – β(eTIVraw_i_ – eTIV_mean_), where β corresponds to the slope of the regression line of each respective brain structure regressed on eTIV. To reduce variance in the brain regions for the latent profile analyses, we converted each measure to cm^3^. These adjusted regions were used in latent profile analyses. MRI field strength was used as a covariate in survival analyses. Creation of ROIs was conducted separately in ADNI and CBAS.

#### Sociodemographic, health, and cognitive variables

We included several factors to describe pairwise differences between the profiles. These other variables were not used in the detection of the latent brain atrophy profiles through the latent profile analysis. Rather, they were included to differentiate the brain atrophy profiles across a range of factors that may also relate to dementia risk. Sociodemographic characteristics included age and sex. Health factors included depressive symptoms (measured with the Geriatric Depression Scale [GDS] 15-item version^30^), because depressive symptoms and cognitive impairment may be interrelated,^31^ and apolipoprotein E (*APOE*) genotype (at least one ε4 allele versus none). For the survival analyses, these variables served as covariates.

Additional sociodemographic characteristics included years of education and occupational position. Occupational position was measured with the International Standard Classification of Occupations (ISCO-08).^32^ Occupation codes are: 1 = managers, 2 = professionals, 3 = technicians and associate professionals, 4 = clerical support workers, 5 = services and sales workers, 6 = skilled agricultural, forestry and fishery workers, 7 = craft and related trades workers, 8 = plant and machine operators and assemblers, and 9 = elementary occupations (i.e., occupations that require simple and routine tasks, often with much physical effort). Armed forces occupations were coded as missing due to the varied nature of military occupations. ISCO coding was available in CBAS. For ADNI, RA and MW completed ISCO coding based on participants’ self-reported main lifetime occupation. Coding was done individually initially with an original agreement of 91.3%. Disagreements were resolved during a consensus meeting between the two coders. Occupational position was coded as missing if participants reported an occupation not represented in the ISCO-08 coding scheme (e.g., housewife) or if they listed armed forces as their main lifetime occupation (see above). ISCO codes were reverse coded for analyses. Years of education and occupational position were only included to describe differences between the latent brain atrophy profiles.

Cognition was measured with three domains: global cognition, executive functioning, and memory. These measures of baseline cognition were included as additional covariates in the survival analyses. *Global cognition* was measured through the Mini Mental State Examination (MMSE)^33^ in both ADNI and CBAS.

In ADNI, *executive functioning* is a composite score identified via item response theory^34^ including: Category Fluency–vegetables and animals, Trail Making Test (TMT) Parts A and B, Digit Span backwards, Wechsler Adult Intelligence Scale-Revised (WAIS-R) Digit Symbol Substitution, and Clock Drawing (specifically, correctly drawing the circle, symbol, numbers, hands, and time). *Memory* is a composite score identified through factor analysis^35^ including: the Rey Auditory Verbal Learning Test (RAVLT) trials 1–5, interference trial, immediate recall, delayed recall, and recognition; the Alzheimer's Disease Assessment Schedule—Cognition Subscale including three learning trials of a word list, delayed word recall, and a word recognition task (present and absent); the MMSE words ball, flag, and tree; and Logical Memory Immediate and Delayed. Both executive functioning and memory are z-scores.

In CBAS, the *executive functioning* domain (Cronbach's α = .82) also comprised measures of attention, psychomotor speed, and working memory and was assessed with the Category Fluency Test–vegetables and animals,^36^ TMT Parts A and B,^37^ Digit Span backwards and the Digit Symbol Coding Test from the WAIS-R,^38^ and the Clock Drawing Test (Cohen scoring system^39^). Participants who did not complete TMT Part A in 180 s or Part B in 300 s were scored as 181 and 301 s, respectively. TMT Parts A and B were reverse coded for analyses, with higher scores indicating better performance. *Memory* (Cronbach's α=.92) was assessed with the RAVLT–Immediate Recall (Sum of Trials 1–5) and Delayed Recall,^40^ the Brief Visuospatial Memory Test–Revised Immediate Recall (Sum of Trials 1–3) and Delayed Recall,^41^ the Uniform Data Set Logical Memory Immediate and Delayed Recall Test,^42^ and the Rey-Osterrieth Complex Figure Test Recall.^43^ For both executive functioning and memory in CBAS, each of the neuropsychological tests was first standardized with z-scores then averaged to create composite domain scores. Finally, we also include participants’ baseline clinical diagnosis (i.e., normal cognition [ADNI only], SCD, MCI).

### Statistical analyses

#### Characterizing the latent profiles

We used latent profile analysis (LPA) in MPlus.^44^ This is an advanced, flexible approach frequently used in the relevant literature^45,46^ that aims to uncover underlying subgroups within a larger sample. LPA is considered methodologically superior to other clustering techniques because (1) it bases class membership on the presence of an underlying latent variable, rather than minimizing distances between members of a group as done in other clustering techniques, and (2) it is a modeled-based technique that provides fit statistics, allowing for determining the best fitting model.^45,46^ LPA was conducted using baseline structural MRI volumetry of the ROIs. Four steps were completed to determine the latent classes: (1) problem definition, (2) model specification, (3) model estimation, and (4) model selection and interpretation.^47^ LPA was conducted separately in ADNI and CBAS using Mplus Version 8.3^44^ which uses full information maximum likelihood estimation to address missing data. While 3–4 classes were expected, we ran models with up to six classes to assess best model fit. The ROIs were converted to cm^3^ to counter excessive variance. Selection of the best-fitting model was done considering the following criteria: having (1) the lowest values (i.e., better model fit^47^) on the Akaike Information Criterion (AIC), the Bayesian Information Criterion (BIC), and the Adjusted BIC; (2) adequate entropy (>.80; the likelihood participants were assigned to profiles correctly^47^), and (3) a significant Vuong-Lo-Mendell-Rubin likelihood ratio test (VLMR-LRT; *p* < 0.05) which indicates better model fit for particular class number specification versus class number-1 specification.^47^ Interpretability of class solutions was also considered as fit statistics sometimes point to different solutions. The distribution of volumetry in the profiles was graphically displayed with boxplots created with R software Version 4.2.2.^48^

After the volumetry-based profiles were revealed, we assessed differences between the brain atrophy profiles in sociodemographic characteristics, health factors, baseline cognitive abilities, and baseline clinical diagnosis with post-hoc pairwise analyses. These other factors were only used to describe differences between brain atrophy profiles, not in the development of profiles. We conducted Analyses of Variance (ANOVAs), Kruskal-Wallis tests, *t*-tests, or chi-square tests as applicable. With the ANOVAs, we used follow-up Tukey HSD tests, and with the Kruskal-Wallis tests, we used the Dwass-Steel-Critchlow-Fligner multiple comparison procedure to assess pairwise differences between profiles for continuous and binary variables, respectively. We used SAS software, Version 9.4 of the SAS System for Windows (SAS Institute, Cary, NC) to conduct descriptive comparisons across the profiles with missing data dropped in a listwise manner.

#### Latent profiles and incident AD dementia

We used Cox proportional hazards regression models^49^ to assess how the brain atrophy profiles related to incident AD dementia. Visual assessment of the Kaplan-Meier curves indicated that the proportional hazards assumption seems to have been met in both ADNI and CBAS. Survival time was calculated as the time from the first assessment to AD dementia or last assessment. In ADNI, mortality data were available, so competing risks analyses^50^ were run as supplemental analyses. Analyses were run unadjusted (Model 1), adjusted for age, sex, and MRI field strength (Model 2), then adjusted for depressive symptoms and *APOE* ε4 status (Model 3), and finally adjusted for baseline cognition (MMSE, executive functioning, memory; Model 4). Survival analysis uses censoring to account for participant dropout and was conducted with SAS software, Version 9.4 of the SAS System for Windows (SAS Institute, Cary, NC), using procedure PHREG. Significance for all analyses was assessed at *p* < 0.05 (two-tailed).

## Results

### Initial profile creation: profiles in ADNI

**Figure 1.**
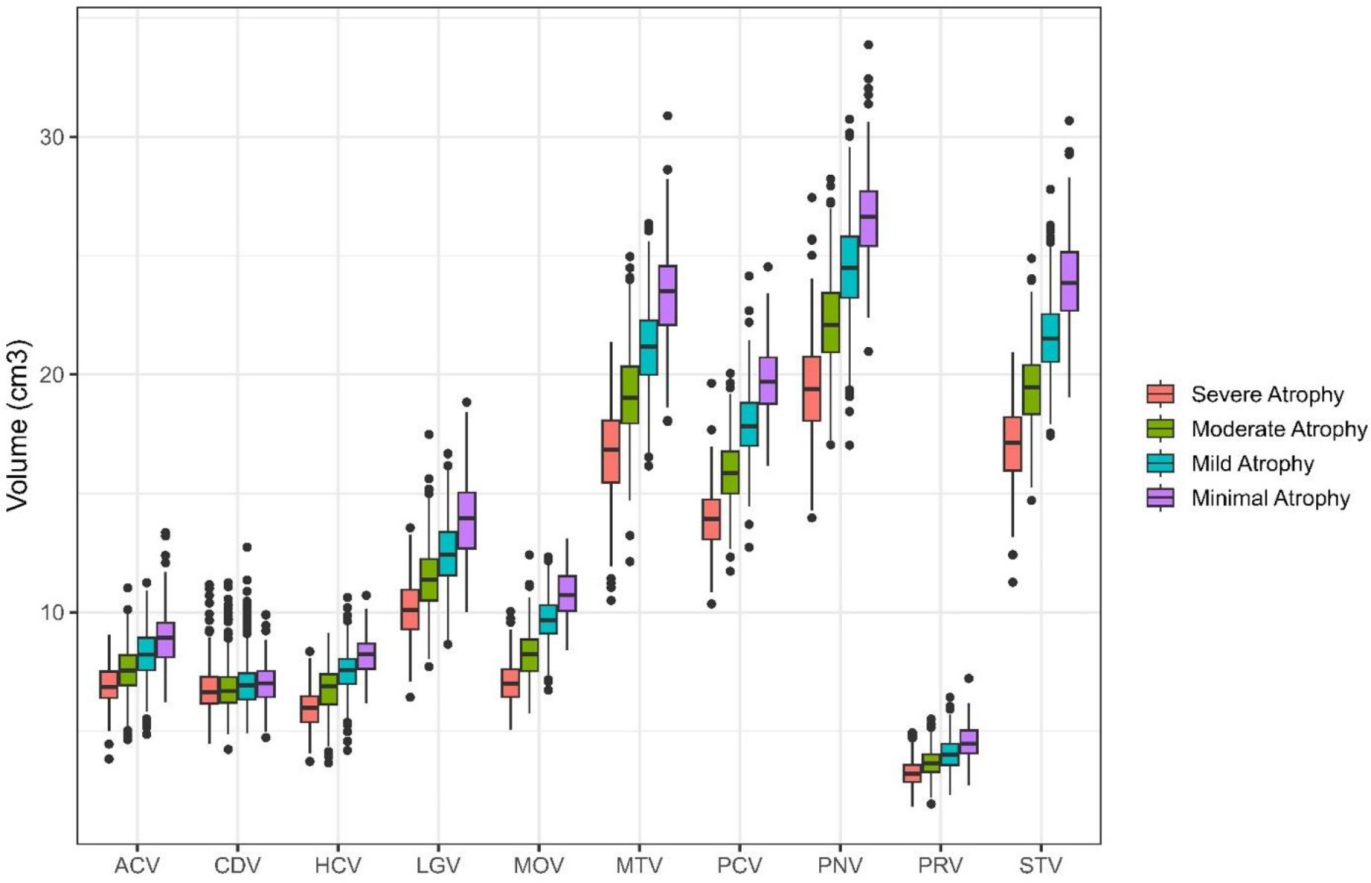
Latent profiles detected in ADNI sample. Four latent profiles were detected in the ADNI sample. Regions of interest are represented on the x-axis with volumes in cm^3^ presented on the y-axis. Each profile was significantly different than each other on all regions of interest except the Caudate, where differences were only found between Moderate Atrophy and Mild and Minimal Atrophy. Minimal Atrophy had the greatest brain volume in all regions of interest, followed by Mild Atrophy, then Moderate Atrophy, with Severe Atrophy having the smallest brain volume. ACV: anterior cingulate cortex volume; CDV: caudate volume; HCV: hippocampal volume; LGV: lingual volume; MOV: medial orbitofrontal volume; MTV: middle temporal volume; PCV: precuneus volume; PNV: precentral volume; PRV: pericalcarine volume; STV: superior temporal volume.

Supplemental Table 1 includes the fit statistics for models run including 1–6 classes. A four-class solution provided the best fit; classes included: Severe Atrophy (253 participants; 14.86%), Moderate Atrophy (567 participants; 33.29%), Mild Atrophy (691 participants; 40.58%), and Minimal Atrophy (192 participants; 11.27%). Figure 1 illustrates the mean brain volumes in cm^3^ of the 10 ROIs for each of the four latent profiles. Each of the profiles followed a similar distribution of brain volumetry, except for the magnitude of volume in most regions. Table 1 presents the results for differences in volumetry from ANOVAs with follow-up Tukey HSD tests for pairwise comparisons between groups. Significant differences were observed across all ROIs among the identified profiles, with the exception of the caudate, which only revealed significant differences between (a) Moderate Atrophy and (b) Mild and Minimal Atrophy. Minimal Atrophy had the greatest brain volume in all ROIs, followed by Mild, Moderate, and Severe Atrophy.

**Table 1.** Differences between profiles in volumetry and participant characteristics in ADNI.

	Severe Atrophy (*n* = 253)		Moderate Atrophy (*n* = 567)		Mild Atrophy (*n* = 691)		Minimal Atrophy (*n* = 192)		
Variable	M ± SD or N (%)		M ± SD or N (%)		M ± SD or N (%)		M ± SD or N (%)		Difference
Region of Interest									
Hippocampus	5.94 ± 0.83		6.75 ± 0.93		7.49 ± 0.83		8.19 ± 0.79		a, b, c, d, e, f
Middle Temporal^†^	16.71 ± 1.90		19.14 ± 1.81		21.15 ± 1.73		23.40 ± 2.13		a, b, c, d, e, f
Superior Temporal^†^	17.04 ± 1.73		19.41 ± 1.55		21.60 ± 1.60		24.00 ± 1.91		a, b, c, d, e, f
Precuneus	13.92 ± 1.32		15.92 ± 1.29		17.91 ± 1.40		19.79 ± 1.47		a, b, c, d, e, f
Anterior Cingulate	6.91 ± 0.80		7.55 ± 0.98		8.24 ± 1.03		8.96 ± 1.21		a, b, c, d, e, f
Medial Orbitofrontal^†^	7.03 ± 0.85		8.20 ± 0.98		9.67 ± 0.89		10.79 ± 0.99		a, b, c, d, e, f
Pericalcarine^†^	3.25 ± 0.54		3.66 ± 0.55		4.03 ± 0.64		4.52 ± 0.74		a, b, c, d, e, f
Precentral^†^	19.47 ± 2.05		22.18 ± 1.88		24.50 ± 1.95		26.69 ± 2.01		a, b, c, d, e, f
Lingual^†^	10.13 ± 1.23		11.38 ± 1.36		12.47 ± 1.38		13.92 ± 1.67		a, b, c, d, e, f
Caudate	6.83 ± 1.06		6.81 ± 0.93		6.98 ± 0.96		7.03 ± 0.91		d, e
Age, years	77.43 ± 6.69		74.47 ± 6.33		71.45 ± 6.61		67.87 ± 5.64		a, b, c, d, e, f
Sex									e, f
Male	143 (56.52%)		286 (50.44%)		346 (50.07%)		129 (67.19%)		
Female	110 (43.48%)		281 (49.56%)		345 (49.93%)		63 (32.81%)		
Depressive Symptoms^†^	1.48 ± 1.42		1.36 ± 1.41		1.27 ± 1.36		0.93 ± 1.26		c, e, f
*APOE* ε4 Status^†^									b
Non-Carrier	129 (51.19%)		322 (57.60%)		416 (62.28%)		114 (61.29%)		
Carrier	123 (48.81%)		237 (42.60%)		252 (37.72%)		72 (38.71%)		
Baseline Diagnosis									a, b, c, d, e, f
Normal Cognition	57 (22.53%)		224 (39.51%)		352 (50.94%)		119 (61.98%)		
Mild Cognitive Impairment	196 (77.47%)		343 (60.49%)		339 (49.06%)		73 (38.02%)		
Progressed to AD Dementia									a, b, c, d, e, f
No	125 (49.41%)		399 (70.37%)		600 (86.83%)		181 (94.27%)		
Yes	128 (50.59%)		168 (29.63%)		91 (13.17%)		11 (5.73%)		
Progressed to Dementia from CN at Baseline	8 (6.25%)		16 (9.52%)		10 (10.99%)		1 (9.09%)		
Progressed to Dementia from MCI at Baseline	120 (93.75%)		152 (90.48%)		81 (89.01%)		10 (90.91%)		
Years of Education	15.79 ± 3.19		16.02 ± 2.68		16.42 ± 2.55		16.85 ± 2.39		b, c, d, e
Occupational Position^†^	5.96 ± 1.80		6.10 ± 1.76		6.27 ± 1.62		6.11 ± 1.89		NS
MMSE	27.28 ± 1.87		28.07 ± 1.73		28.56 ± 1.57		29.02 ± 1.26		a, b, c, d, e, f
Executive Functioning*^,^^†^	−0.11 ± 0.92		0.31 ± 0.80		0.73 ± 0.87		1.11 ± 0.84		a, b, c, d, e, f
Memory*^,^^†^	0.08 ± 0.76		0.42 ± 0.72		0.75 ± 0.70		0.90 ± 0.84		a, b, c, d, e, f

AD: Alzheimer's disease; *APOE* ε4: presence of ɛ4 allele of apolipoprotein E gene; CN: normal cognition; M: mean; MCI: mild cognitive impairment; MMSE: Mini-Mental State Examination; NS: not significant; SD: standard deviation. Volumes are presented in cm^3^ to align with values of these variables in the latent profile analysis conducted. Differences between profiles assessed with one-way analyses of variance (ANOVAs) with follow-up Tukey HSD tests and Kruskal-Wallis tests with the Dwass-Steel-Critchlow-Fligner multiple comparison procedure to assess overall differences among groups on variables with pairwise comparisons between profiles for continuous and binary variables, respectively. ^a^Difference between Severe Atrophy and Moderate Atrophy. ^b^Difference between Severe Atrophy and Mild Atrophy. ^c^Difference between Severe Atrophy and Minimal Atrophy. ^d^Difference between Moderate Atrophy and Mild Atrophy. ^e^Difference between Moderate Atrophy and Minimal Atrophy. ^f^Difference between Mild Atrophy and Minimal Atrophy.

^*^Executive functioning and memory represent averaged domain scores based on standardized (z-scored) neuropsychological tests.

^†^ Missingness ranged from <1% to 6%.

#### Differences between ADNI profiles

Table 1 also describes how the four profiles differ according to other AD risk factors. Participants with Minimal Atrophy were the youngest, followed by participants with Mild, Moderate, and Severe Atrophy. Those with Minimal Atrophy were more frequently men compared to Mild and Moderate Atrophy, which both had an approximately equal distribution of men and women. Participants with Minimal Atrophy had significantly lower depressive symptoms compared to participants with Severe, Mild, and Moderate Atrophy. Participants with Mild Atrophy had fewer *APOE* ε4 carriers compared to participants with Severe Atrophy. All four profiles differed from each other on the three measures of baseline cognition. Participants with Minimal Atrophy were the most cognitively intact (or unimpaired), followed by participants with Mild, Moderate, and Severe Atrophy. Participants with Severe and Moderate Atrophy had lower education than participants with Mild and Minimal Atrophy. There were no significant differences between profiles in occupational position.

#### ADNI profiles and incident AD dementia (Supplemental Table 2)

**Figure 2.**
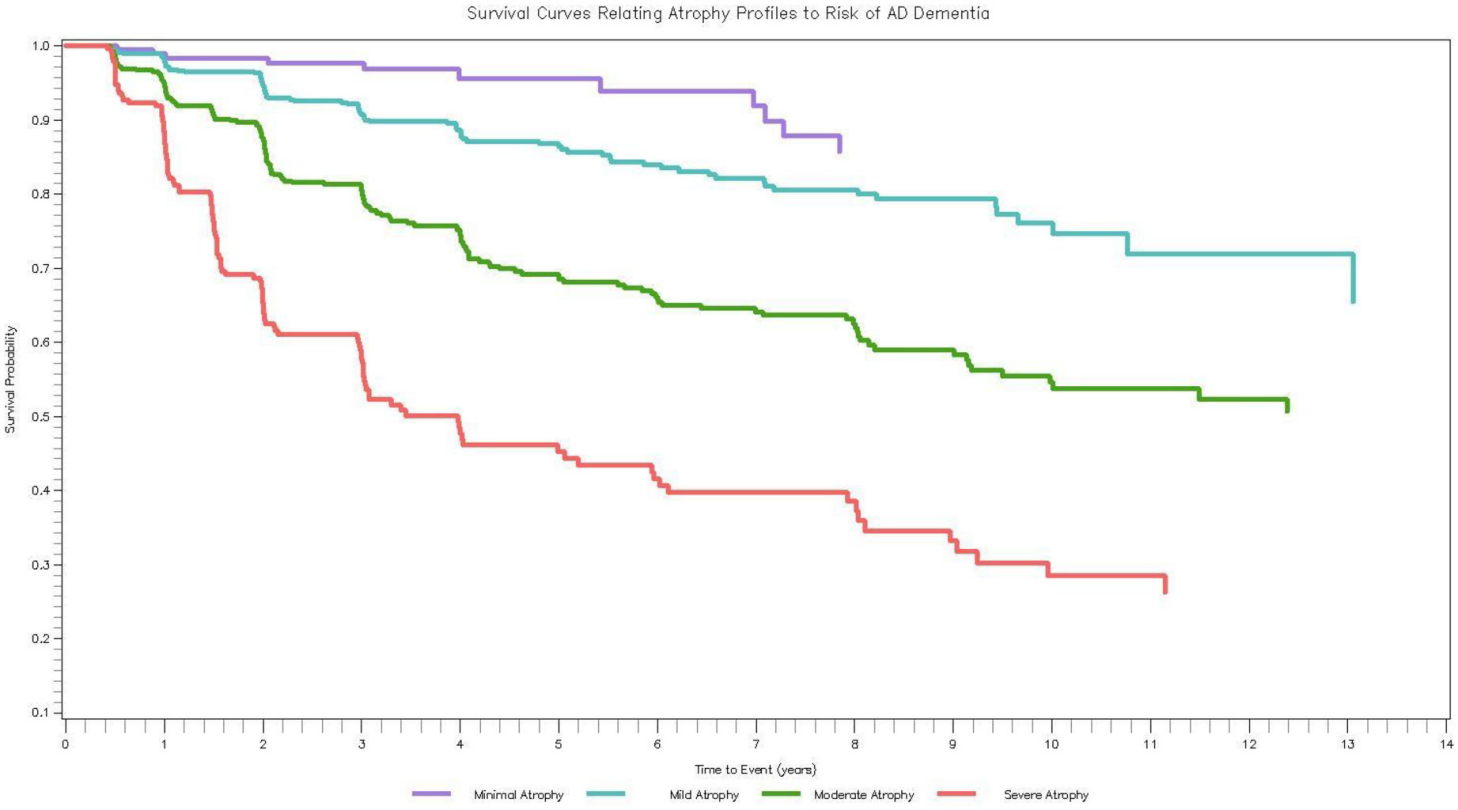
How ADNI profiles relate to incident AD dementia. Survival curves (Kaplan-Meier) of the brain atrophy profiles in relation to incident AD dementia. Average time to dementia progression was 2.97 years (*SD* = 2.52) whereas average time to censoring was 4.85 years (*SD* = 3.55).

In up to 16 years of follow-up, 398 (23.37%) ADNI participants developed AD dementia (including 35 with normal cognition and 363 with MCI at baseline). In the covariate-adjusted model (Model 3), participants with Mild (HR = 3.11, 95% CI [1.43, 6.78]), Moderate (HR = 7.58, 95% CI [3.45, 16.68]), and Severe (HR = 16.95, 95% CI [7.39, 39.86]) Atrophy had a higher risk of incident AD dementia compared to Minimal Atrophy. Controlling for baseline cognition reduced the magnitude of the results for Moderate Atrophy (HR = 3.01, 95% CI [1.35, 6.69]) and Severe Atrophy (HR = 4.80, 95% CI [2.05, 11.25]), and reduced the result for Mild Atrophy to non-significance (HR = 1.90, 95% CI [0.87, 4.18]). Figure 2 contains the Kaplan-Meier survival curves illustrating time to incident AD dementia for each of the brain atrophy profiles. Results from competing risks analyses were consistent with main analyses (Supplemental Table 3).

### Profile validation: profiles in CBAS

**Figure 3.**
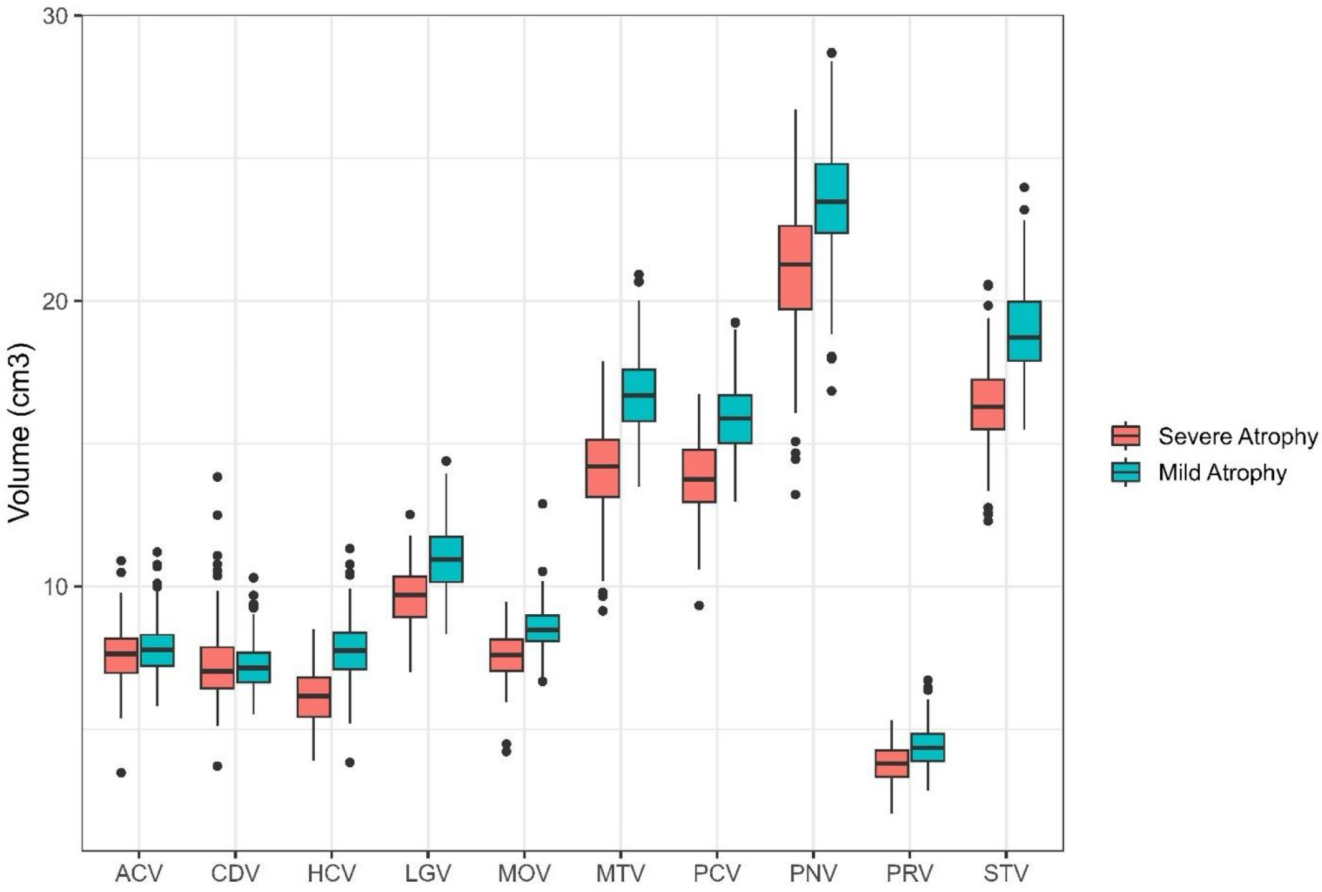
Latent profiles detected in CBAS sample. Two latent profiles were detected in the CBAS sample. Regions of interest are represented on the x-axis with volumes in cm^3^ presented on the y-axis. Both profiles were significantly different than each other on all regions of interest except the Caudate. Mild Atrophy had greater brain volume than Severe Atrophy. ACV: anterior cingulate cortex volume; CDV: caudate volume; HCV: hippocampal volume; LGV: lingual volume; MOV: medial orbitofrontal volume; MTV: middle temporal volume; PCV: precuneus volume; PNV: precentral volume; PRV: pericalcarine volume; STV: superior temporal volume.

Supplemental Table 4 includes the fit statistics for models run with 1–5 classes. The 2-class solution was the best fitting model; classes included: Severe Atrophy (177 participants; 45.97%) and Mild Atrophy (208 participants; 54.03%). Figure 3 illustrates the mean brain volumes in cm^3^ for each of the 10 ROIs for both profiles. Table 2 includes results from *t*-tests comparing volumetry between profiles. Participants with Mild Atrophy had greater brain volume in all ROIs except the caudate region compared to participants with Severe Atrophy. Both profiles followed a similar pattern of brain volumetry across ROIs, indicating differences only in magnitude of volume.

**Table 2.** Differences between profiles in volumetry and participant characteristics in CBAS.

	Severe Atrophy (*n* = 177)		Mild Atrophy (*n* = 208)		
Variable	M ± SD or N (%)		M ± SD or N (%)		Difference
Region of Interest					
Hippocampus	6.13 ± 0.96		7.70 ± 1.01		**<0**.**001**
Middle Temporal	14.05 ± 1.67		16.72 ± 1.41		**<0**.**001**
Superior Temporal	16.34 ± 1.43		18.92 ± 1.53		**<0**.**001**
Precuneus	13.82 ± 1.31		15.94 ± 1.32		**<0**.**001**
Anterior Cingulate	7.57 ± 0.99		7.84 ± 0.92		**0**.**005**
Medial Orbitofrontal	7.61 ± 0.85		8.57 ± 0.78		**<0**.**001**
Pericalcarine	3.77 ± 0.64		4.37 ± 0.70		**<0**.**001**
Precentral	21.01 ± 2.28		23.52 ± 1.91		**<0**.**001**
Lingual	9.64 ± 1.05		10.97 ± 1.14		**<0**.**001**
Caudate	7.26 ± 1.32		7.22 ± 0.83		0.752
Age, years	74.70 ± 6.94		67.95 ± 6.70		**<0**.**001**
Sex					0.555
Male	73 (41.24%)		92 (44.23%)		
Female	104 (58.76%)		116 (55.77%)		
Depressive Symptoms^†^	3.27 ± 2.57		3.63 ± 3.02		0.220
*APOE* ε4 Status^†^					**0**.**013**
Non-Carrier	89 (50.28%)		127 (61.06%)		
Carrier	81 (45.76%)		68 (32.69%)		
Baseline Diagnosis					**<0**.**001**
Subjective Cognitive Decline	25 (14.12%)		84 (40.38%)		
Mild Cognitive Impairment	152 (85.88%)		124 (59.62%)		
Progressed to AD Dementia					**<0**.**001**
No	97 (54.80%)		178 (85.58%)		
Yes	80 (45.20%)		30 (14.42%)		
Progressed to Dementia from SCD at Baseline	1 (1.25%)		2 (6.67%)		
Progressed to Dementia from MCI at Baseline	79 (98.75%)		28 (93.33%)		
Years of Education^†^	14.78 ± 3.05		15.20 ± 3.35		0.199
Occupational Position^†^	4.86 ± 1.71		4.96 ± 1.63		0.562
MMSE^†^	25.91 ± 2.92		27.98 ± 1.94		**<0**.**001**
Executive Functioning^*,^^†^	−0.39 ± 0.61		0.31 ± 0.64		**<0**.**001**
Memory*	−0.53 ± 0.70		0.31 ± 0.77		**<0**.**001**

AD: Alzheimer's disease; *APOE* ε4: presence of ε4 allele of apolipoprotein E gene; M: mean; MCI: mild cognitive impairment; MMSE: Mini-Mental State Examination; SCD: subjective cognitive decline; SD: standard deviation. Volumes are presented in cm^3^ to align with values of these variables in the latent profile analysis conducted. Differences between profiles assessed with *t*-tests or chi-square tests for continuous and binary variables, respectively. Bolded values indicate significant effects at *p* < 0.05.

^*^Executive functioning and memory represent average domain scores based on standardized (z-scored) neuropsychological tests.

† Missingness ranged from <1% to 12%.

#### Differences between CBAS profiles

Table 2 also describes how the two profiles differ in other AD risk factors. Participants with Mild Atrophy were significantly younger than those with Severe Atrophy, about similar in terms of distribution by sex and depressive symptomatology, less likely to be *APOE* ε4 carriers, had fewer participants with MCI, and had better cognitive scores than those with Severe Atrophy. Participants with Severe Atrophy and Mild Atrophy did not differ in years of education or occupational position.

#### CBAS profiles and incident AD dementia (Supplemental Table 5)

**Figure 4.**
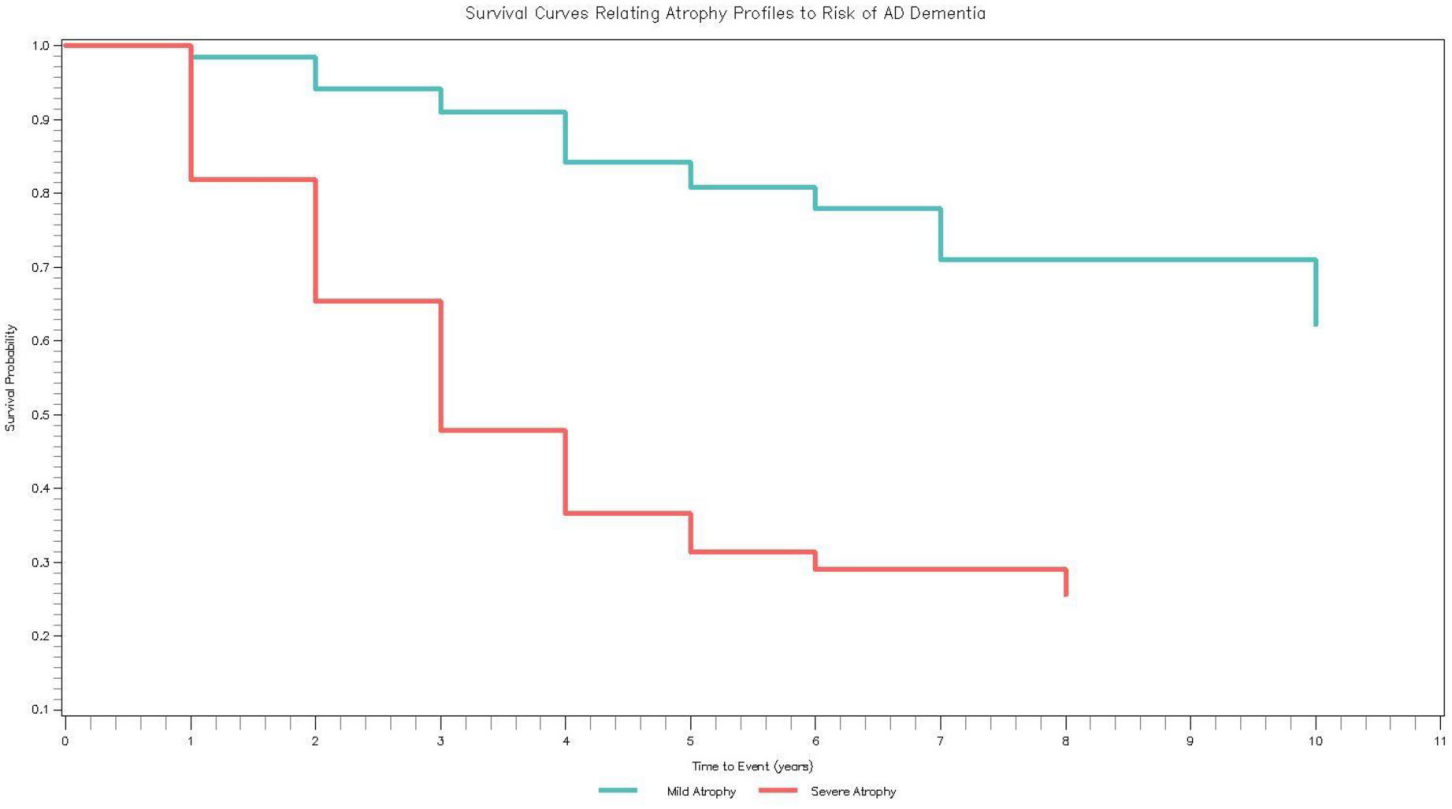
How CBAS profiles relate to incident AD dementia. Survival curves (Kaplan-Meier) of the brain atrophy profiles in relation to incident AD dementia. Average time to dementia progression was 2.72 years (*SD* = 1.76) whereas average time to censoring was 3.29 years (*SD* = 2.80).

In up to 14 years of follow-up, 110 (28.57%) CBAS participants developed AD dementia (3 with SCD and 107 with MCI at baseline). In the covariate-adjusted model (Model 3), participants with Severe Atrophy had an increased risk of incident AD dementia compared to participants with Mild Atrophy (HR = 3.51, 95% CI [2.14, 5.77]). This result was reduced to non-significance with the addition of baseline cognition (HR = 1.65, 95% CI [0.96, 2.81]). Figure 4 contains the Kaplan-Meier survival curves illustrating time to incident AD dementia for both brain atrophy profiles.

### Qualitative comparison between samples

As described previously, the two datasets yielded four- and two-profile solutions, not fully replicating patterns across studies. However, the profiles followed similar patterns in each of the ROIs, with profiles differentiating only on magnitude of atrophy. Here, we qualitatively compare the two most similar profiles that emerged in both datasets, Mild and Severe Atrophy, also summarized in Table 3. Both datasets yielded smaller volumes in the Severe Atrophy profile compared to the Mild Atrophy profile in all ROIs except the caudate region. In both datasets, participants with Severe Atrophy were older than participants with Mild Atrophy, but participants in both groups in ADNI were slightly older than participants in both groups in CBAS. Neither sample exhibited differences between the proportion of men and women in the profiles, but there were more men in ADNI than CBAS. The profiles did not differ in depressive symptomatology in either ADNI or CBAS; however, depressive symptoms were slightly higher for CBAS than ADNI participants, which is common in clinic-based samples. The profiles differed in *APOE* ε4 carrier status, such that the Severe Atrophy profile was evenly split between carriers and non-carriers and the Mild Atrophy profile had fewer carriers than non-carriers–these proportions were nearly identical in both datasets.

**Table 3.** Similarities and differences between severe and mild atrophy profiles in ADNI and CBAS.

Variable	ADNI	CBAS
Volumetry	Smaller brain volumetry in Severe vs. Mild Atrophy for all regions but the caudate region.	Smaller brain volumetry in Severe vs. Mild Atrophy for all regions but the caudate region.
*Sociodemographic Characteristics*		
Age	Severe Atrophy (*M* = 77.43, *SD* = 6.69) older than Mild Atrophy (*M* = 71.45, *SD* = 6.61).	Severe Atrophy (*M* = 74.70, *SD* = 6.94) older than Mild Atrophy (*M* = 67.95, *SD* = 6.70).
Sex	No significant difference in proportion of men in the profiles for Severe (56.52%) vs. Mild (50.07%) Atrophy.	No significant difference in proportion of men in the profiles for Severe (41.24%) vs. Mild (44.23%) Atrophy.
Years of Education	Fewer years of education for Severe (*M* = 15.79, *SD* = 3.19) vs. Mild (*M* = 16.42, *SD* = 2.55) Atrophy.	No significant difference in years of education for Severe (*M* = 14.78, *SD* = 3.05) vs. Mild (*M* = 15.20, *SD* = 3.35) Atrophy.
Occupational Position	No significant difference in occupational position for Severe (*M* = 5.96, *SD* = 1.80) vs. Mild (*M* = 6.27, *SD* = 1.62) Atrophy.	No significant difference in occupational position for Severe (*M* = 4.86, *SD* = 1.71) vs. Mild (*M* = 4.96, *SD* = 1.63) Atrophy.
*Health Factors*		
Depressive Symptoms	No significant difference in depressive symptoms for Severe (*M* = 1.48, *SD* = 1.42) vs. Mild (*M* = 1.27, *SD* = 1.36) Atrophy.	No significant difference in depressive symptoms for Severe (*M* = 3.27, *SD* = 2.57) vs. Mild (*M* = 3.63, *SD* = 3.02) Atrophy.
*APOE* ε4 Status	Approximately even split between carriers (48.81%) and non-carriers (51.19%) in Severe Atrophy; fewer carriers (37.72%) than non-carriers (62.28%) in Mild Atrophy.	Approximately even split between carriers (45.76%) and non-carriers (50.28%) in Severe Atrophy; fewer carriers (32.69%) than non-carriers (61.06%) in Mild Atrophy.
*Clinical Factors*		
Baseline Diagnosis	More participants with MCI (77.47%) than normal cognition (22.53%) in Severe Atrophy; approximately even distribution of participants with normal cognition (50.94%) and MCI (49.06%) in Mild Atrophy.	Many more participants with MCI (85.88%) than SCD (14.12%) in Severe Atrophy; slightly more participants with MCI (59.62%) than SCD (40.38%) in Mild Atrophy.
Progression to AD Dementia	Approximately even distribution of progressing (50.59%) vs. not (49.41%) for Severe Atrophy; fewer progressing (13.17%) than not (86.83%) for Mild Atrophy.	Approximately even distribution of progressing (45.20%) vs. not (54.80%) for Severe Atrophy; fewer progressing (14.42%) than not (85.58%) for Mild Atrophy.
*Baseline Cognition*		
Global Cognition	Poorer global cognition for Severe (*M* = 27.28, *SD* = 1.87) vs. Mild (*M* = 28.56, *SD* = 1.57) Atrophy.	Poorer global cognition for Severe (*M* = 25.91, *SD* = 2.92) vs. Mild (*M* = 27.98, *SD* = 1.94) Atrophy.
Executive Functioning	Poorer executive functioning for Severe (*M* = −0.11, *SD* = 0.92) vs. Mild (*M* = 0.73, *SD* = 0.87) Atrophy.	Poorer executive functioning for Severe (*M* = −0.39, *SD* = 0.61) vs. Mild (*M* = 0.31, *SD* = 0.64) Atrophy.
Memory	Poorer memory for Severe (*M* = 0.08, *SD* = 0.76) vs. Mild (*M* = 0.75, *SD* = 0.70) Atrophy.	Poorer memory for Severe (*M* = −0.53, *SD* = 0.70) vs. Mild (*M* = 0.31, *SD* = 0.77) Atrophy.

AD: Alzheimer's disease; *APOE* ε4: presence of ε4 allele of apolipoprotein E gene; M: mean; MCI: mild cognitive impairment; SCD: subjective cognitive decline; SD: standard deviation.

Additionally, participants in the Severe Atrophy profile were more likely to have MCI than normal objective cognition (normal cognition and SCD) in both samples, though there was a slightly higher proportion of participants with MCI in CBAS than ADNI. In the Mild Atrophy profile, ADNI was evenly split between participants with MCI and normal cognition, whereas there were slightly more participants with MCI than SCD in CBAS. In both ADNI and CBAS, approximately 50% of participants with Severe Atrophy and approximately 15% of participants with Mild Atrophy progressed to AD dementia during the follow-up. In ADNI, participants with Severe Atrophy had fewer years of education than those with Mild Atrophy. The profiles did not differ in education in CBAS. Years of education were lower overall for CBAS compared to ADNI.

In neither ADNI nor CBAS were there differences between the profiles in occupational position; occupational position overall was slightly lower in CBAS compared to ADNI. Finally, baseline cognition was poorer in the Severe Atrophy profile compared to the Mild Atrophy profile in both ADNI and CBAS. Global cognition was lower in both profiles for CBAS compared to ADNI, with the Mild Atrophy profile in CBAS scoring about the same as the Severe Atrophy profile in ADNI. For executive functioning and memory, participants in the Severe Atrophy profile performed about 1SD lower than the Mild Atrophy profile in both datasets. Overall, the Severe and Mild Atrophy profiles in ADNI and CBAS relate to other factors that impact AD dementia risk very similarly, even with some differences between the samples in age, depressive symptoms, education, and global cognition.

## Discussion

We aimed to identify brain atrophy profiles in two datasets from geographically, sociodemographically, and culturally distinct regions, thus carrying out an immediate replication of our results from ADNI with the distinct CBAS sample. Specifically, we compared the profiles across a relatively comprehensive battery of presumed factors related to AD dementia risk and assessed how the profiles related to incident AD dementia. In ADNI, four brain atrophy profiles emerged characterized by increasing levels of atrophy, whereas in CBAS two profiles emerged. Contrary to expectations, the atrophy patterns reflected differences only in the magnitude of volume that appeared consistent across ROIs. Generally, as expected, participants with less atrophy had the most favorable characteristics (Tables 1 and 2).

The differences in the final profile solutions for the two samples could be due to several reasons. First, although both samples include participants along the clinical spectrum (normal objective cognition to mild cognitive impairment), participants in CBAS were recruited through a memory clinic and may have been farther along in clinical impairment compared to the ADNI sample. Indeed, nearly 72% of the CBAS sample included participants with a baseline diagnosis of MCI versus 56% in the ADNI sample. Further, the remaining participants in CBAS had a diagnosis of SCD, indicating that the patient or an informant detected signs of initial cognitive decline, whereas ADNI participants had normal cognition.

Second, ADNI and CBAS come from distinct geographic, cultural, and sociodemographic environments (the U.S. and Czech Republic) and participants likely had starkly different life course experiences. Further, within the U.S., there is greater racial/ethnic and sociocultural diversity compared to the Czech Republic, which is more homogenous, which may point to additional within-sample variability in ADNI to detect distinct profiles. As the most recent Lancet report suggests 45% of dementia prevalence could be reduced through targeting modifiable risk factors^51^ and with the contribution of structural and social determinants to dementia,^52^ differences in contextual factors between the two samples could have also contributed to the different profile solutions. Finally, the CBAS sample was smaller than the ADNI sample, which could have limited the number of detectable profiles. Future work should consider these factors when assessing patterns of atrophy across different geographic and sociocultural regions.

We also found that participants from profile clusters with more extensive atrophy had higher incident AD dementia risk compared to those in profiles with less atrophy even when controlling for variables known to relate to AD dementia risk. This was observed as a dose-response effect in the ADNI sample. In this context, it is important to note that the magnitude of AD dementia risk associated with each atrophy profile was greater than the magnitude of effects for *APOE* ε4 status, age, and depressive symptomatology in both samples (Supplemental Tables 2 and 5), indicating that volumetry has high clinical relevance in recognizing AD dementia risk in the preclinical and prodromal stages. When additionally controlling for baseline cognition, the magnitude of associations for Moderate and Severe Atrophy profiles in ADNI was reduced but remained significant, whereas effects for Mild Atrophy in ADNI and Severe Atrophy in CBAS were attenuated to non-significance. These results suggest that some Atrophy profiles do provide distinct information past baseline cognitive abilities. However, our lack of differentiation in how atrophy profiles related to AD dementia risk among CBAS participants when additionally controlling for baseline cognition may be due to sample differences. Indeed, global cognition was similar between the Mild Atrophy profile in CBAS and the Severe Atrophy profile in ADNI, providing additional support for the potential of limited clinical variability and/or more extensive clinical severity in CBAS.

Our data-driven approach to categorizing brain atrophy profiles allows for detection of heterogeneity across ROIs.^12^ However, we observed that variability in patterns of atrophy did not contribute to profile classification. Instead, profile classification seemed to be driven predominantly by magnitude of atrophy. This is despite our use of 10 ROIs known to be relevant to AD dementia onset and progression.^6^ Magnitude of atrophy may be a more robust parameter than pattern of atrophy, especially when considering findings across distinct regions and diverse populations. Specifically, pattern of atrophy can change over time (i.e., atrophy across different regions can accelerate and decelerate; it is not a parameter changing linearly), and various stages of the same disease can show varying patterns of atrophy at different stages. Additionally, distinct samples may have different proportions of various clinical AD manifestations (e.g., dysexecutive AD versus typical AD) which presumes different proportions of certain atrophy patterns. Finally, there are geographic, cultural, and sociodemographic differences between samples that may differentially influence brain atrophy, suggesting that magnitude, rather than pattern, may be a more stable parameter across groups.

Notably, the severity of atrophy related to higher risk of incident AD dementia regardless of baseline cognitive abilities or clinical diagnoses, which underscores the importance of assessing global atrophy. AD is characterized by progressive, diffuse neurodegeneration. In the stage of MCI or dementia, atrophy is widespread across the cortex, not confined to one region. Consistent with this, AD patients typically exhibit widespread, symmetrically distributed^53^ cortical volume loss on MRI. This diffuse atrophy reflects the cumulative disease burden, which often correlates with clinical severity. Thus, the degree of global atrophy effectively captures the overall neurodegenerative burden of AD, offering robust diagnostic signal even if specific regional patterns vary between individuals.

Further, emphasizing global atrophy in diagnosis aligns with current biomarker frameworks and can enhance clinical practice. The latest research criteria (AT(N) framework) include MRI measures of atrophy as a core indicator of neurodegeneration (the “N”) in AD^15^ which is also a part of revised criteria.^54^ Marked global cortical atrophy on MRI is a strong marker of underlying neurodegeneration, analogous in its significance to elevated tau biomarkers.^55^ In clinical practice, an MCI patient whose MRI shows disproportionate whole-brain volume loss (for their age) is likely experiencing a pathologic neurodegenerative process, such as the process that underlies incipient AD. While such global atrophy is not entirely specific to AD (i.e., other neurodegenerative disorders can also cause diffuse atrophy), when considered in combination with characteristic cognitive symptoms, diagnostic confidence can be increased. Prior studies have found that an aggregate measure of brain atrophy can discriminate AD from healthy aging with accuracy comparable to classic regional markers like hippocampal volume.^56^ For example, in the cohort reported by Ingala and colleagues (2022), a simple visual rating of global cortical atrophy (the GCA scale) achieved diagnostic performance (AUC ≈ 0.84) on par with medial temporal atrophy ratings for separating patients with AD from controls.^56^ Therefore, characterizing global atrophy via MRI in clinical examinations among those in preclinical and prodromal phases of neurodegenerative diseases like AD can help identify individuals who may be most at risk of future impairment as well as provide an opportunity to facilitate support for these individuals.

Even though our two samples have notable recruitment (research- versus clinic-based), geographic (U.S. versus Czech Republic), sociodemographic, and cultural differences and have participants who may be considered farther along in clinical progression in one (CBAS including participants with SCD) versus the other (ADNI including participants with normal cognition), we found similar, though not exactly the same, profiles in both datasets. Specifically, we identified both the Mild and Severe Atrophy profiles in both datasets that yielded similarities across samples in other risk factors for AD dementia. The higher proportion of *APOE* ε4 carriers found in the Severe Atrophy profiles in both samples may be due to a direct association between *APOE* ε4 and brain volume^57^ or through *APOE* ε4's association with the AD neurodegenerative process.^58^ Our design was somewhat different than past work by including participants with normal objective cognition and MCI simultaneously, whereas others have focused on participants with normal cognition^9^ or MCI^6,10^ only. However, since AD exists along a continuum and with mismatch sometimes occurring between amount of pathology and clinical diagnoses^15–18^ (i.e., older adults exhibiting different levels of cognitive resilience to neuropathology^59–61^), by including participants across the AD continuum, we were able to directly assess how extent of atrophy, rather than clinical diagnosis, relates to AD dementia risk. Within each of the profiles, there was a combination of participants with both normal objective cognition and MCI. Thus, the volumetric information across brain regions seems to be only one part of diagnostic status rather than the sole, or even main, contributor to it.

We used MRI regions identified previously^6^ as the paradigm for the current project, which was based on ADNI data. Our results were somewhat consistent with these previous findings with three volume-based profiles differentiated by cognitive scores decreasing across profiles with increasing atrophy. However, previously,^6^ one other profile was found such that the hippocampus was selectively impaired separately from the other ROIs and had poorer executive functioning only—this pattern diverged from the other profiles that suggested greater atrophy across all ROIs related to poorer cognitive functioning. In our study, we found that cognitive outcomes mirrored the volumetry-based profiles more “cleanly”. Reasons for discrepancies could include differences in sample composition or size. Unlike the prior study^6^ that included only participants with MCI in their LPA, we included participants with normal cognition, preclinical (SCD), and prodromal (MCI) cognitive statuses. It could be that differential atrophy patterns may crystalize later in the disease progression. Indeed, characterization of heterogeneous neuropathology/atrophy patterns was initially done among individuals with prevalent dementia^13^ with more recent work attempting to characterize heterogeneity earlier in the disease process.^7^ Further, our sample size was larger (1703 versus 696) which may have contributed to differences. Despite the somewhat different findings between studies, both point to the presence of biological variability among participants without dementia that are related to cognitive outcomes.

Our work is consistent with other research using data-driven methodologies to detect brain atrophy or neuropathology profiles. One study^9^ used cluster analysis to detect profiles based on cerebrospinal fluid and MRI measures in older adults with normal cognition. They also found a graded distribution of neuropathology, with three subtypes having normal, somewhat impaired, and severely impaired imaging and biomarker profiles. Participants in the severely impaired profile had neuropathology as extensive as participants with MCI or dementia.^9^ In a study focused on participants with MCI,^10^ four neuropathology profiles based on MRI and AD biomarkers were detected that had gradually increasing neuropathology. Aside from the clearly differentiated “healthiest” biomarker cluster which had all AD biomarkers similar to individuals with normal cognition and the “unhealthiest” biomarker cluster with AD biomarkers similar to individuals with AD dementia, the two intermediary clusters that were suggestive of early AD dementia, based on their similar beta-amyloid levels, differed such that one had less extensive tau pathology in conjunction with more extensive structural (MRI) pathology.^10^ In both studies,^9,10^ profiles with more severe atrophy had worse cognitive outcomes, which agrees with our findings. Finally, consistent with prior work,^6,10^ we found that profiles reflective of more severe atrophy were associated with increased AD dementia risk compared to a relatively “healthy” imaging profile. These results highlight the importance of assessing brain volumetry in clinical assessments to help identify who may be at most risk of cognitive decline.

### Strengths, limitations, and future directions

Strengths of the study include: (1) classifying brain atrophy profiles in participants without dementia across two datasets from geographically, culturally, and sociodemographically distinct regions, offering the ability to validate profiles across datasets and unique opportunities for qualitative comparisons; (2) including only volumetry in the profile creation which prevented sociodemographic characteristics or cognitive abilities from driving profile detection; (3) describing differences between profiles found in several other AD risk factors with differences emerging as would be expected based on levels of atrophy; and (4) using survival analysis to assess how the profiles related to incident AD dementia adjusted for common confounds.

Limitations included: (1) using cross-sectional MRI data which prevents differentiation between disease-related changes or premorbid developmental differences as contributors to brain volumetry; (2) considering only 10 brain regions in analyses, which may have limited the emergence of profiles that have subtly varying atrophy patterns in other regions; (3) not including AD biomarkers in the creation of profiles which may not fully represent biological heterogeneity patterns that may exist in beta-amyloid or tau; and (4) not having loss to follow up information available for most participants (84%) in CBAS, so competing risks analyses could not be conducted. However, considering mortality as a competing risk in ADNI yielded similar results to main analyses. Finally, both datasets are racially and ethnically homogeneous (i.e., most reported being White), so future work should include more diverse samples for increased generalizability.

Future research should: (1) consider latent trajectory analysis to measure brain atrophy patterns over time; (2) include additional brain regions to determine whether other profiles exist (since as a data-driven methodology, the emergence of profiles is based on the data available for profile formation); and (3) consider AD biomarkers in profile formation. Additionally, we used a data-driven rather than hypothesis-driven (i.e., the Murray-Dickson criteria^62^ which classifies individuals based on their ratios of cortical to medial temporal lobe atrophy and includes four typical atrophy patterns: typical AD, limbic predominant, hippocampal sparing, and minimal atrophy) approach and found divergent patterns. Future research should directly compare these methodologies to determine which is best at identifying the most clinically relevant profiles. Although we used a data-driven approach to characterize brain atrophy profiles, plausibly allowing for different patterns of atrophy across brain regions to appear within the latent subgroups, our results revealed differences only in magnitude of atrophy, with different groups found across the two datasets. To facilitate cross-national comparisons, future work could characterize levels of atrophy similarly (e.g., according to volume percentiles) across diverse cohorts, which would also allow for direct comparisons between atrophy levels and dementia risk across studies. Finally, future research should consider within-group heterogeneity to better understand how various factors across the life course may contribute to differential brain health in older adulthood and subsequent dementia risk.

### Conclusion

We identified four distinct brain atrophy profiles in ADNI and two in CBAS, with differences driven primarily by the magnitude, rather than the pattern, of atrophy. While profiles were not fully replicable across datasets, their qualitative similarity underscores the generalizability of these findings across geographically and culturally distinct cohorts. The extent (magnitude) of atrophy significantly predicted incident AD dementia, independent of major risk factors such as *APOE* ε4 status, age, depressive symptoms, and baseline cognition, highlighting its clinical relevance. Incorporating volumetry into diagnostic and risk assessment protocols could enhance the early identification of AD dementia.

## Supplemental Material

sj-docx-1-alz-10.1177_13872877251371734 - Supplemental material for Differential MRI atrophy profiles and incident dementia: A cross-national comparison Supplemental material, sj-docx-1-alz-10.1177_13872877251371734 for Differential MRI atrophy profiles and incident dementia: A cross-national comparison by Monica E Walters, Brent J Small, Ross Andel, Ondrej Lerch, Hana Horakova , Victor A Molinari, Jason L Salemi, Martin Vyhnalek, Zuzana Nedelska, Jakub Hort and for the Alzheimer's Disease Neuroimaging Initiative in Journal of Alzheimer's Disease
