# Supplementary material for: Differential MRI atrophy profiles and incident dementia: A cross-national comparison
Source: J Alzheimers Dis. 2025 Sep 3;107(4):1539–54. doi: 10.1177/13872877251371734 (PMC12550212; doi:10.1177/13872877251371734)
Supplement: sj-docx-1-alz-10.1177_13872877251371734 - Supplemental material for Differential MRI atrophy profiles and incident dementia: A cross-national comparison [file sj-docx-1-alz-10.1177_13872877251371734.docx]

**Supplemental Material**

**Differential MRI atrophy profiles and incident dementia: A cross-national comparison**

| **Supplemental Table 1**. Fit statistics of different class sizes from latent profile analysis in ADNI sample. | | | | | | |
| --- | --- | --- | --- | --- | --- | --- |
| **Number of Classes** | **AIC** | **BIC** | **Adjusted BIC** | **Entropy** | **VLMR-LRT (*p*)** | **Participants Per Class** |
| **1** | 63,697.47 | 63,806.27 | 63,742.73 | N/A | N/A | 1,703 |
| **2** | 59,243.88 | 59,412.52 | 59,314.04 | 0.865 | <0.001 | 750; 953 |
| **3** | 58,151.03 | 58,379.52 | 58,246.09 | 0.815 | 0.017 | 400; 784; 519 |
| **4** | 57,669.62 | 57,957.95 | 57,789.57 | 0.816 | 0.004 | 253; 691; 567; 192 |
| **5** | 57,490.36 | 57,838.53 | 57,635.21 | 0.784 | 0.036 | 258; 552; 214; 546; 133 |
| **6** | 57,344.75 | 57,752.76 | 57,514.49 | 0.769 | 0.229 | 77; 290; 470; 510; 121; 235 |
| AIC: Akaike Information Criteria; BIC: Bayesian Information Criteria; VLMR-LRT: Vuong-Lo-Mendell-Rubin likelihood ratio test. Smaller values for the AIC, BIC, and Adjusted BIC indicate better model fit. Entropy > 0.80 is considered good. The VLMR-LRT indicates whether a model with c classes fits the data better than a model with c-1 classes (significant *p*-value). Entropy and the VLMR-LRT are not available in the one-class solution since there is no class separation to determine and no fewer classes with which to compare this model. Although the five-class solution had lower values for the AIC, BIC, and Adjusted BIC, and seemed to provide a better fit than the four-class solution (VLMR-LRT: *p*=0.036), the entropy level was lower than the accepted limit (0.78). A six-class solution was also tested but had worse fit than the five-class solution (VLMR-LRT: *p*=0.229) and lower entropy (0.77). | | | | | | |

| **Supplemental Table 2.**  Survival analysis results relating brain atrophy profiles to incident AD dementia in ADNI. | | | | | | | | | | | |
| --- | --- | --- | --- | --- | --- | --- | --- | --- | --- | --- | --- |
|  | **Model 1^a^** | |  | **Model 2^b^** | |  | **Model 3^c^** | |  | **Model 4^d^** | |
| **Variable** | **HR** | **95% CI** |  | **HR** | **95% CI** |  | **HR** | **95% CI** |  | **HR** | **95% CI** |
| **Mild Atrophy** | **2.25** | **1.20, 4.20** |  | **3.28** | **1.51, 7.12** |  | **3.11** | **1.43, 6.78** |  | 1.90 | 0.87, 4.18 |
| **Moderate Atrophy** | **5.14** | **2.79, 9.45** |  | **8.53** | **3.89, 18.70** |  | **7.58** | **3.45, 16.68** |  | **3.01** | **1.35, 6.69** |
| **Severe Atrophy** | **11.88** | **6.42, 22.00** |  | **21.43** | **9.41, 48.83** |  | **16.95** | **7.39, 39.86** |  | **4.80** | **2.05, 11.25** |
| **MRI Field Strength** | --- | --- |  | 1.05 | 0.87, 1.26 |  | 1.03 | 0.85, 1.24 |  | 1.02 | 0.84, 1.23 |
| **Age, Years** | --- | --- |  | **0.97** | **0.96, 0.99** |  | 0.99 | 0.97, 1.00 |  | **0.98** | **0.97, 1.00** |
| **Sex, Female** | --- | --- |  | **0.75** | **0.61, 0.92** |  | **0.74** | **0.59, 0.91** |  | 1.24 | 0.99, 1.55 |
| ***APOE* ε4 Carrier** | --- | --- |  | --- | --- |  | **2.86** | **2.31, 3.54** |  | **1.81** | **1.44, 2.26** |
| **Depressive Symptoms** | --- | --- |  | --- | --- |  | **1.15** | **1.08, 1.24** |  | 1.05 | 0.97, 1.13 |
| **MMSE** | --- | --- |  | --- | --- |  | --- | --- |  | 0.97 | 0.91, 1.04 |
| **Executive Functioning** | --- | --- |  | --- | --- |  | --- | --- |  | **0.75** | **0.65, 0.87** |
| **Memory** | --- | --- |  | --- | --- |  | --- | --- |  | **0.21** | **0.17, 0.26** |
| HR: hazard ratio; CI: confidence interval. Minimal Atrophy was the reference category (= 0) for each Atrophy profile (=1). MRI field strength was coded as 1.5 or 3 (Tesla). Sex was coded as female = 1, male = 0. *APOE* ε4 Carrier: carrier of ε4 allele of apolipoprotein E gene (= 1) versus non-Carrier (= 0). Depressive symptoms were measured with the Geriatric Depression Scale (GDS) 15-item version (higher scores = more depressive symptoms). MMSE: Mini-Mental State Examination. Bolded values indicate significant effects at *p* < 0.05. ^a^*n* = 1,703. ^b^*n* = 1,578. ^c^*n* =1,539. ^d^*n* = 1,535. | | | | | | | | | | | |

| **Supplemental Table 3.** Competing risks analyses relating brain atrophy profiles to incident AD dementia in ADNI. | | |
| --- | --- | --- |
| **Variable** | **HR** | **95% CI** |
| **Mild Atrophy** | 1.93 | 0.83, 4.47 |
| **Moderate Atrophy** | **2.92** | **1.23, 6.89** |
| **Severe Atrophy** | **4.57** | **1.81, 11.52** |
| **MRI Field Strength** | 0.98 | 0.80, 1.20 |
| **Age, Years** | **0.97** | **0.96, 0.99** |
| **Sex, Female** | **1.27** | **1.01, 1.60** |
| ***APOE* ε4 Carrier** | **1.72** | **1.37, 2.18** |
| **Depressive Symptoms** | 1.04 | 0.96, 1.12 |
| **MMSE** | 0.98 | 0.91, 1.06 |
| **Executive Functioning** | **0.75** | **0.64, 0.88** |
| **Memory** | **0.22** | **0.18, 0.28** |
| *n*=1,535. 94 censored participants died before a potential AD dementia diagnosis; a competing risk of death was included for these participants in these analyses. HR: hazard ratio; CI: confidence interval. Minimal Atrophy was the reference category (=0) for each Atrophy profile (=1). MRI field strength was coded as 1.5 or 3 (Tesla). Sex was coded as female = 1, male = 0. *APOE* ε4 Carrier = carrier of 𝜀4 allele of apolipoprotein E gene (=1) versus non-Carrier (=0). Depressive symptoms were measured with the Geriatric Depression Scale (GDS) 15-item version (higher scores = more depressive symptoms). MMSE: Mini-Mental State Examination. Bolded values indicate significant effects at *p* < 0.05. | | |

| **Supplemental Table 4.** Fit Statistics of Different Class Sizes from Latent Profile Analysis in CBAS Sample. | | | | | | |
| --- | --- | --- | --- | --- | --- | --- |
| **Number of Classes** | **AIC** | **BIC** | **Adjusted BIC** | **Entropy** | **VLMR-LRT (*p*)** | **Participants Per Class** |
| **1** | 13,224.67 | 13,303.75 | 13,240.30 | N/A | N/A | 385 |
| **2** | 12,494.34 | 12,616.89 | 12,518.53 | 0.831 | <0.001 | 177; 208 |
| **3** | 12,368.77 | 12,534.81 | 12,401.55 | 0.798 | 0.300 | 125; 197; 63 |
| **4** | 12,291.19 | 12,500.71 | 12,332.55 | 0.823 | 0.493 | 146; 38; 173; 28 |
| **5** | 12,243.77 | 12,496.78 | 12,293.72 | 0.797 | 0.050 | 42; 69; 80; 166; 28 |
| AIC: Akaike Information Criteria; BIC: Bayesian Information Criteria; VLMR-LRT: Vuong-Lo-Mendell-Rubin likelihood ratio test. Smaller values for the AIC, BIC, and Adjusted BIC indicate better model fit. Entropy > 0.80 is considered good. The VLMR-LRT indicates whether a model with c classes fits the data better than a model with c-1 classes (significant *p*-value). Entropy and the VLMR-LRT are not available in the one-class solution since there is no class separation to determine and no fewer classes with which to compare this model. | | | | | | |

| **Supplemental Table 5.** Survival Analysis Results Relating Brain Atrophy Profiles to Incident AD Dementia in CBAS. | | | | | | | | | | | |
| --- | --- | --- | --- | --- | --- | --- | --- | --- | --- | --- | --- |
|  | **Model 1^a^** | |  | **Model 2^b^** | |  | **Model 3^c^** | |  | **Model 4^c^** | |
| **Variable** | **HR** | **95% CI** |  | **HR** | **95% CI** |  | **HR** | **95% CI** |  | **HR** | **95% CI** |
| **Severe Atrophy** | **4.90** | **3.21, 7.50** |  | **3.89** | **2.43, 6.23** |  | **3.51** | **2.14, 5.77** |  | 1.65 | 0.96, 2.81 |
| **MRI Field Strength** | --- | --- |  | **2.22** | **1.20, 4.12** |  | **2.20** | **1.10, 4.39** |  | **2.97** | **1.45, 6.06** |
| **Age, Years** | --- | --- |  | **1.04** | **1.01, 1.07** |  | **1.05** | **1.02, 1.08** |  | 1.01 | 0.97, 1.04 |
| **Sex, Female** | --- | --- |  | 1.46 | 0.99, 2.15 |  | **1.53** | **1.02, 2.30** |  | 1.26 | 0.82, 1.94 |
| ***APOE* ε4 Carrier** | --- | --- |  | --- | --- |  | **2.14** | **1.40, 3.29** |  | 0.82 | 0.49, 1.35 |
| **Depressive Symptoms** | --- | --- |  | --- | --- |  | 0.98 | 0.90, 1.06 |  | 0.95 | 0.88, 1.03 |
| **MMSE** | --- | --- |  | --- | --- |  | --- | --- |  | 0.93 | 0.86, 1.02 |
| **Executive Functioning** | --- | --- |  | --- | --- |  | --- | --- |  | **0.58** | **0.38, 0.89** |
| **Memory** | --- | --- |  | --- | --- |  | --- | --- |  | **0.24** | **0.15, 0.37** |
| HR: hazard ratio; CI: confidence interval. Mild Atrophy was the reference category (=0) for Severe Atrophy (=1). MRI field strength was coded as 1.5 or 3 (Tesla). Sex was coded as female = 1, male = 0. *APOE* ε4 Carrier: carrier of ε4 allele of apolipoprotein E gene (=1) versus non-Carrier (=0). Depressive symptoms were measured with the Geriatric Depression Scale (GDS) 15-item version (higher scores = more depressive symptoms). MMSE: Mini-Mental State Examination. Bolded values indicate significant effects at *p* < 0.05. ^a^*n* = 385. ^b^*n* = 384. ^c^*n* =351. | | | | | | | | | | | |
